# Inter-Species Comparative Analysis of Components of Soluble Sugar Concentration in Fleshy Fruits

**DOI:** 10.3389/fpls.2016.00649

**Published:** 2016-05-19

**Authors:** Zhanwu Dai, Huan Wu, Valentina Baldazzi, Cornelis van Leeuwen, Nadia Bertin, Hélène Gautier, Benhong Wu, Eric Duchêne, Eric Gomès, Serge Delrot, Françoise Lescourret, Michel Génard

**Affiliations:** ^1^EGFV, Bordeaux Sciences Agro, INRA, Université de BordeauxVillenave d’Ornon, France; ^2^INRA, UR1115, Plantes et Systèmes de Culture HorticolesAvignon, France; ^3^Bordeaux Sciences Agro, ISVV, UMR 1287 EGFV Villenave d’Ornon, France; ^4^Institute of Botany – Chinese Academy of SciencesBeijing, China; ^5^INRA, UMR 1131 SVQVColmar, France

**Keywords:** dilution, fruit metabolism, grape, peach, sugar importation, tomato

## Abstract

The soluble sugar concentration of fleshy fruit is a key determinant of fleshy fruit quality. It affects directly the sweetness of fresh fruits and indirectly the properties of processed products (e.g., alcohol content in wine). Despite considerable divergence among species, soluble sugar accumulation in a fruit results from the complex interplay of three main processes, namely sugar import, sugar metabolism, and water dilution. Therefore, inter-species comparison would help to identify common and/or species-specific modes of regulation in sugar accumulation. For this purpose, a process-based mathematical framework was used to compare soluble sugar accumulation in three fruits: grape, tomato, and peach. Representative datasets covering the time course of sugar accumulation during fruit development were collected. They encompassed 104 combinations of species (3), genotypes (30), and growing conditions (19 years and 16 nutrient and environmental treatments). At maturity, grape showed the highest soluble sugar concentrations (16.5–26.3 g/100 g FW), followed by peach (2.2 to 20 g/100 g FW) and tomato (1.4 to 5 g/100 g FW). Main processes determining soluble sugar concentration were decomposed into sugar importation, metabolism, and water dilution with the process-based analysis. Different regulation modes of soluble sugar concentration were then identified, showing either import-based, dilution-based, or import and dilution dual-based. Firstly, the higher soluble sugar concentration in grape than in tomato is a result of higher sugar importation. Secondly, the higher soluble sugar concentration in grape than in peach is due to a lower water dilution. The third mode of regulation is more complicated than the first two, with differences both in sugar importation and water dilution (grape vs. cherry tomato; cherry tomato vs. peach; peach vs. tomato). On the other hand, carbon utilization for synthesis of non-soluble sugar compounds (namely metabolism) was conserved among the three fruit species. These distinct modes appear to be quite species-specific, but the intensity of the effect may significantly vary depending on the genotype and management practices. These results provide novel insights into the drivers of differences in soluble sugar concentration among fleshy fruits.

## Introduction

Fresh fruits (such as grape, tomato, and peach) and their processed products (e.g., wine from grape) have a major economical importance. Fresh fruits also play an essential role in the composition of a healthy diet. The composition of fruits largely determines their sensory properties, their nutritional value, and hence, consumer preference and the final profit for fruit growers. Among other compounds, soluble sugars are one of the major determinants of fruit quality. They directly impact the sweetness and taste of fresh fruits and provide precursors for the synthesis of other quality-related compounds, such as organic acids, anthocyanins, and aroma compounds. They affect alcohol content after fermentation in processed products (e.g., wine). For example, consumers prefer peaches with a high (∼9.5–10%) value of total soluble solids (TSSs, mainly soluble sugars) rather than fruits with a lower TSS (<8%; Grechi et al., 2008). On the other hand, a too high soluble sugar content (TSS over 30%) in grape leads to a high alcohol level in wines, which may be detrimental for the perception of wine quality and the health of wine consumers (Duchêne and Schneider, 2005). Therefore, modulating fruit sugar concentration to an attractive and desirable level for the consumers has scientific interest and agronomical relevance.

Soluble sugar concentration as well as sugar composition show large variations across species (Coombe, 1976). For example, grape has a very high soluble sugar concentration (∼2 mmol/gFW) compared to other fleshy fruits (Coombe, 1992), while peach and tomato has, respectively, moderate (∼0.4 mmol/gFW; Quilot et al., 2004) and low (∼0.15 mmol/gFW) soluble sugar concentration (Prudent et al., 2011; Biais et al., 2014). The form of soluble sugars stored in fruits can be hexoses (glucose and fructose) dominated with trace sucrose (most of grape and tomato varieties) or sucrose dominated with moderate levels of hexoses (peach and few specific varieties of grape and tomato) and low levels of sorbitol (peach; Desnoues et al., 2014).

Soluble sugar concentration in fruit is the result of several processes. First, photoassimilates are imported into the fruit, following phloem unloading. Different phloem unloading mechanisms exist (Lalonde et al., 2003; Kühn and Grof, 2010) and their coordination follows specific developmental patterns depending on the species (Ruan and Patrick, 1995; Zhang et al., 2006; Zanon et al., 2015). Second, imported photoassimilates are metabolized in apoplasm, symplasm or vacuole and partly used to synthesize cell walls, organic acids or storage compounds (e. g. starch in tomato). Although sugar metabolism shares similar reaction pathways associated with common enzymes, such as sucrose synthase (SuSy), sucrose phosphate synthase (SPS) and invertase (INV), specificities exist for individual species depending on the nature of accumulated soluble sugars (e.g., sorbitol for peach). Moreover, the evolution of enzymes activities over fruit development may significantly differ among species, whereas it appears pretty stable among genotypes of the same species (Biais et al., 2014; Desnoues et al., 2014). Last but not least, dilution by water also plays an important role in determining the concentration of soluble sugars (Génard et al., 2014) and it is known to be largely affected by environmental conditions or management practices. For example, a negative correlation is usually found between sugar content and irrigation levels (Kobashi et al., 2000; Castellarin et al., 2007; Sadras and McCarthy, 2007; Ripoll et al., 2016). Therefore, any difference in soluble sugar concentration among species or among genotypes within a given species may result from the different contributions of sugar importation, sugar metabolism, and/or dilution, during fruit development.

Considering that the basic processes determining soluble sugar concentration are similar, multispecies comparison may help to understand whether the main control levers of soluble sugar concentration are species-specific or follow a species-overarching manner. However, multispecies comparison among fruits is largely hampered by (a) the complex nature of sugar accumulation as affected by the genotype x environment interactions and (b) the lack of proper tools to integrate information into a common framework to make comparable the results from different species. Recently, Klie et al. (2014) identified some conserved dynamics of metabolic processes across species during fruit development with a generalized principal component approach (STATIS). STATIS can capture similarities and differences between multiple tables containing metabolite data during different fruit development and ripening stages, providing a way of multispecies comparison of metabolism in fruits (Klie et al., 2014). However, as other statistical analysis approaches, STATIS analyzes the metabolite profiles but cannot provide indications on biological processes that may affect these metabolite profiles.

Process decomposition may serve as an alternative framework for multispecies comparison. It can dissect a complex trait into processes more physiological relevant and stable over changing environments (Bertin et al., 2010). A number of frameworks indeed have been developed that describe the temporal evolution of soluble sugar concentration within the fruit and have been used to evaluate the contributions of sugar importation, metabolism and water dilution on changes in soluble sugar concentration, under contrasted environment or genotypes, for a panel of species (Génard et al., 2003; Quilot et al., 2004; Dai et al., 2009; Prudent et al., 2011). However, inter-species comparison by using this approach has never been attempted so far.

Inspired by these studies, we propose here to use process-based decomposition as a tool for multispecies comparison. Based on experimental data, the contribution of sugar importation, metabolism and water dilution on soluble sugar concentration was computed all over fruit development and used to analyze the drivers causing the inter-species variability in soluble sugar concentration, and to identify similarities and differences among three fruit species. A particular attention was also devoted to investigate genotypic variability and the effect of environment and management practices on the regulation of soluble sugar concentration.

## Materials and Methods

### Data sources

Developmental profiles of fruit flesh fresh weight (FW), dry weight (DW), and soluble sugar concentration (SC) were collected for three fruit species (grape, tomato, and peach) from both published literatures and unpublished data (**Supplementary Table S1**). In total, there were 104 different sugar accumulation profiles, covering 30 genotypes and various growing conditions (19 years and 17 nutrient and environmental treatments; **Supplementary Table S1**). Grape and peach datasets were mainly focused on the second rapid growth phase, ranging from 30 to 140 days after flowering (DAF), with 5–12 sampling points at regular intervals of 7–15 days in each profile. Tomato datasets covered the full fruit development stages, ranging from 5 to 70 DAF, with 7–14 sampling points at regular intervals of 5–10 days. Crop load (an agricultural term describing the ratio between leaf surface and number of fruits for a fruit tree) treatments that can modulate the source-sink relationships were imposed to some genotypes of peach and tomato. In addition, the truss (or the bunch) position of fruits within a plant was also included in the analysis for tomato. At least three biological replicates were used at each sampling date. The three fruit species were chosen for the analysis because (1) their data are collected within a long-term collaboration network where protocols and analysis were rather standardized with various genotypes and years; (2) these three fruit species are representative of drupes, berry and fleshy fruits as well as non-climacteric and climacteric fruits, making their comparison meaningful from a biological perspective.

Flesh FW was measured by weighing whole fruit, and then seed weight was excluded for peach (Génard et al., 2003); jelly and seed were excluded for tomato in Prudent et al. (2009) but whole fruit were considered in other studies of cherry tomato and tomato (Bertin et al., 2009); an average proportion of 12.5% of seed and skin weight in grape berry was excluded (Dai et al., 2009). Flesh DW was obtained from FW by subtracting flesh water content (WC). The WC of peach and tomato were obtained by drying a pre-weighed piece of fresh fruit. For grape, the WC was empirically determined as a function of soluble sugar concentration (Garcia de Cortazar-Atauri et al., 2009). Soluble sugars were measured either with enzymatic method (Génard et al., 2003; Prudent et al., 2009) or HPLC method (Wu et al., 2012). For some grape samples, TSSs (^o^Brix) were determined using a PAL-1 portable electronic refractometer and an empirical relationship was then applied to transform ^o^Brix into hexose concentration (OIV, 2009). Total soluble sugar concentration was obtained by summing up all the sugar forms accumulated in the fruit, including sucrose, glucose, fructose, and sorbitol as described by Quilot et al. (2004). To make data comparable, all FW and DW were expressed in gram, and soluble sugar concentration in g sugar/100 gFW.

### Process-Based Comparative Approach

As described in Quilot et al. (2004), carbon arrives into the fruit as sugars, via the phloem. In the flesh, part of this flow of carbon is used as substrates for respiratory pathways. The remaining carbon is used partly for soluble sugar synthesis and partly for synthesis of other carbohydrate compounds (e.g., starch, acids, structural carbohydrates, and proteins). Accordingly, the variation in soluble sugar concentration (SC) in the fruit results from the balance among three different processes, the net sugar import rate from the plant to the fruit (import rate – respiration rate, *u*), the rate of metabolic consumption of soluble sugars to synthesize other compounds (*m*) and the rate of dilution (*d*) as the volume of the fruit increases:

$$\frac{dSC}{dt}=u\left(t\right)+m\left(t\right)+d\left(t\right)$$

The net sugar uptake rate *u* (g/100 g/day) can be calculated directly from dry mass variation of the fruit as done by Génard et al. (2003):

$$u=\frac{100_{\gamma DW}}{\gamma_{sugar}FW}\frac{dDW}{dt}$$

where γ_DW_ represents the carbon concentration of the flesh (gC per gram of dry mass) and γ_sugar_ is the mean carbon content of sugars (gC/g sugars).

In an analogous way, the dilution rate *d* describes the soluble sugar concentration loss caused by fruit volume increases and can be derived from fresh mass variation as in Génard et al. (2003):

$$d=\frac{SC}{FW}\frac{dFW}{dt}$$

Note that both *u*, *m*, and *d* components can be time, genotype and environment dependent.

By integrating all over fruit development, the overall contri bution of each process, for a given genotype and environment, can be defined at fruit maturity as:

$$U=\int_{t_{0}}^{t_{m}}u(t)dt,\;M=\int_{t_{0}}^{t_{m}}m(t)dt,\;D=\int_{t_{0}}^{t_{m}}d(t)dt$$

By definition,

$$\Delta SC=SC(t_{m})-{SC}_{0}=U+M+D$$

where SC(t_0_) and SC(t_m_) are the total soluble sugar concentrations at the beginning of experiment and at maturity, respectively.

To calculate the three components (U, M, and D), observed developmental curves of FW, DW, and SC were fitted by local regression to compute a daily value. $\frac{dFW}{dt}$ and $\frac{dDW}{dt}$ were then calculated by derivation of daily FW and DW. Once U and D determined from Eq. 4, the total metabolic component M can be computed from the difference M = SC(t_m_)+SC(t_0_)-U-D, providing an estimate of the overall sugar turnover during fruit development.

### Statistical Analysis

The data analysis was conducted using the *R* Statistical Computing Environment (R Development Core Team, 2010). The local regression of FW, DW, and SC were obtained with the “loess” function and the derivation of FW and DW with the “diff” function. The differential equations were numerically integrated using the Euler method with a 1-day time step.

Statistical methods suitable for unbalanced one-way factorial dataset are needed to determine if one variable is significantly different among fruit species. To this end, “gao_cs” function of “nparcomp” package was applied to conduct a non-parametric multiple comparison (Baudrit et al., 2015). Principal component analysis (PCA) was performed on mean-centered and scaled data with “dudi.pca” function of “ade4” package (Dray and Dufour, 2007), in order to compare three drivers of soluble sugar concentration among fruit species. PCA was first made by using the three drivers of sugar concentration (namely the U, M, D), and then FW, DW, and SC at maturity were projected as non-active variables. In this way, one can assess the discriminations of different fruit species, genotypes, and growth conditions by the three components and compare the prediction quality of the PCs identified from the active dataset in relation to the non-active dataset.

## Results

### Fruit Size and Soluble Sugar Concentration at Maturity

**FIGURE 1:**
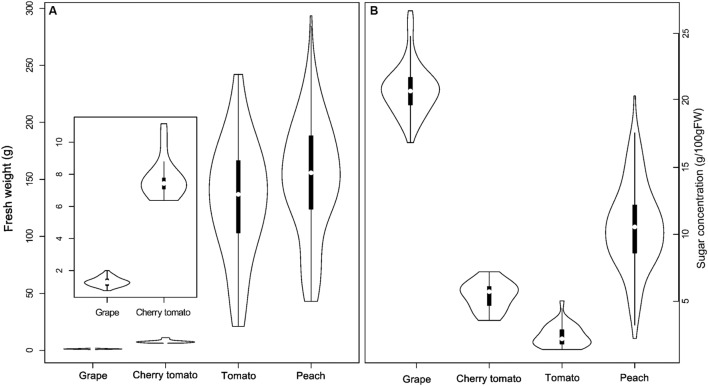
**Distribution of fruit fresh weight (FW; **A**) and soluble sugar concentration **(B)** in grape, cherry tomato, tomato, and peach at maturity.** White points represent median of FW and soluble sugar concentration of a given fruit species in the dataset. The violin shape represents the distribution of the variables in each fruit species.

Based on a pre-analysis, cherry tomato was found to behavior differently from normal tomato in both final sugar concentration and contributions of the three main components. Therefore, cherry tomato was treated separately in the following sections, although it belongs to the same species as tomato. The FW of fruits at maturity varied among fruit species, showing peach ≥ tomato > cherry tomato > grape, in the studied dataset (**Figure 1**). Fruit species also showed a large diversity in soluble sugar concentration at maturity, with grape having the highest soluble sugar concentrations (16.5 to 26.3 g/100 g FW), followed by peach (2.2 to 20 g/100 g FW), cherry tomato (3.5 to 6.1 g/100 g FW), and tomato (1.4 to 5 g/100 g FW; **Figure 1**). Comparing FW and soluble sugar concentration, it is clear that the smallest fruit species (grape) had the highest soluble sugar concentration. However, peach weight is higher than cherry tomato and tomato but it had a higher concentration of soluble sugars.

### Dynamics of Fruit Growth and Soluble Sugar Concentration Over Fruit Development

**FIGURE 2:**
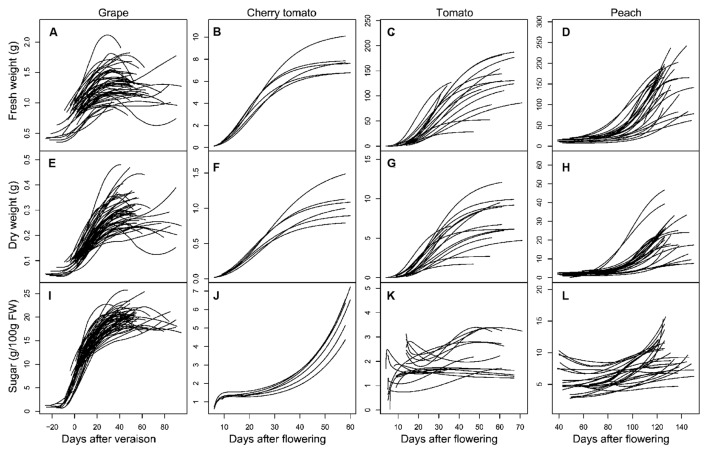
**Developmental profiles of FW **(A–D)**, dry weight (DW; **E–H**), and soluble sugar concentration **(I–L)** in grape **(A,E,I)**, cherry tomato **(B,F,J)**, tomato **(C,G,K)**, and peach **(D,H,L)** fruits.** All curves are smoothed fits of the observed data points using the “loess” function in R software (R Development Core Team, 2010).

It is well-known that grape and peach fruits have a double-sigmoid growth curve (DeJong and Goudriaan, 1989; Coombe and McCarthy, 2000), while tomato fruit has a single-sigmoid growth curve (Bertin et al., 2009). In the present dataset, developmental profiles of grape and peach covered mainly the second rapid growth stage, while those of tomato covered almost the full developmental stages (**Figure 2**). As a consequence, during the studied period, fresh and DWs of all the three fruits exhibited similar dynamics: remaining at low level at beginning, then increasing sharply, and reaching a plateau around maturity (**Figures 2A–H**). Despite these similarities, soluble sugar concentration showed large differences in their developmental dynamics. Soluble sugar concentration of grape increased strongly from veraison on, and reached a plateau approaching maturity (**Figure 2I**); Cherry tomato showed a continuous and exponential increase in soluble sugar concentration up to the maturity (**Figure 2J**); tomato and peach had much smaller fluctuations of sugar accumulation, even exhibited decreases in soluble sugar concentration over fruit development (**Figures 2K,L**).

### Contributions of Sugar Importation, Metabolism, and Dilution on Soluble Sugar Concentration among Different Fruit Species

**FIGURE 3:**
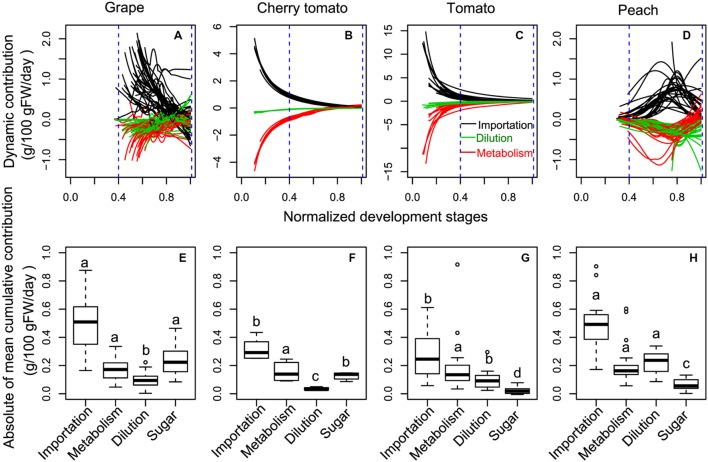
**The dynamic **(A–D)** and the absolute value of mean cumulative **(E–H)** contributions of sugar importation, sugar metabolism, and water dilution on sugar accumulation in grape, cherry tomato, tomato, and peach during the late fruit development stages.** Sugar represents the mean increment of sugar concentration during the targeted period **(E–H)**. To make the developmental profiles comparable among fruits, development stages were normalized with flowering to be 0 and maturity to be 1. The absolute value of cumulative contributions were calculated over the period from 40% maturity to 100% maturity (blue dashed lines), and then divided by the duration (days) of the chosen period for each condition. Different letters for a given component in different fruits **(E–H)** indicate a significant difference, based on non-parametric multiple comparison for unbalanced one-way factorial designs in R.

To gain insights into the potential drivers underlying the differences in soluble sugar concentration among the three fruit species (**Figure 1**), developmental profiles in **Figure 2** were subjected into the process-based analysis to decompose soluble sugar concentration into three processes, namely sugar importation, metabolism and water dilution (**Figure 3**). Moreover, development stages were normalized, with flowering to be 0 and maturity to be 1, to make the developmental profiles comparable among fruit species (**Figures 3A–D**). After this normalization, it is clear that most of the developmental profiles spanned from 40% maturity to 100% maturity for the three fruit species, and therefore, cumulative contribution of the three processes was calculated over this period (**Figures 3E–H**). To take into account the variation in duration between 40 and 100% maturity, the cumulative contribution was further divided by the duration (days) of the chosen period for each condition (**Figures 3E–H**).

Over the considered developmental stages, peach showed a distinct dynamics of sugar importation, metabolism, and water dilution, in comparison with those of grape, cherry tomato, and tomato (**Figures 3A–D**). In grape, cherry tomato and tomato, higher sugar importation, metabolism, and water dilution were observed at early developmental stages, and they simultaneously approached to zero at maturity (**Figures 3A–C**). On the other hand, the three processes of peach were low around 40% of maturity, then peaked around 75% of maturity, and approached to zero thereafter (**Figure 3D**). Interestingly, the metabolism changed from negative value to positive value around maturity, particularly for cherry tomato (**Supplementary Figure S1B**) and in some cases for the other fruits (**Supplementary Figures S1A,C,D**). In addition, dilution also changed from negative to positive value around maturity for grape (**Supplementary Figure S1A**).

**FIGURE 4:**
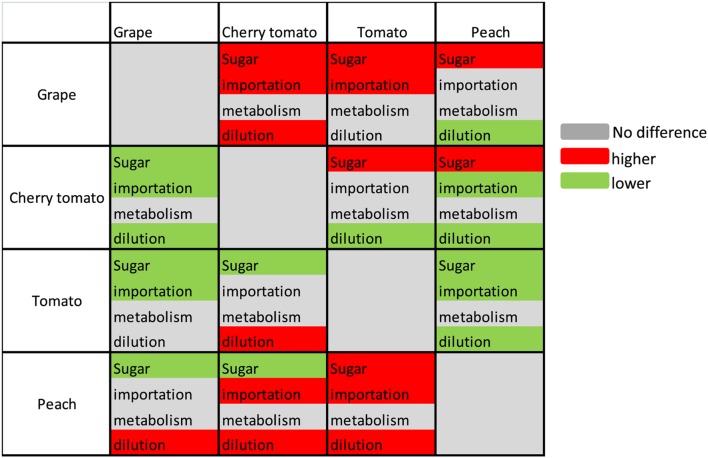
**Summary of the differences among fruit species regarding soluble sugar concentration and of its components, sugar importation, sugar metabolism, and water dilution.** Different colors indicate the difference of each criterion between the fruit at row and the fruit at column, with red for higher, green for lower, and gray for no difference. Sugar represents the mean increment of sugar concentration during the targeted period.

The absolute values of mean cumulative contributions of sugar importation, metabolism, and water dilution were of the same order of magnitude regardless of the species over the period of 40% maturity to 100% maturity (**Figures 3E–H**). The sugar importation was always the most important component with a contribution 2–3 times that of metabolism or dilution. Sugar importation was higher in grape and peach than in cherry tomato and tomato. Metabolism did not show significant differences among the three fruit species. Water dilution was the highest in peach, followed by grape and tomato, and lowest in cherry tomato. Based on these statistical results, the modes causing differences in soluble sugar concentration among fruit species were then summarized in **Figure 4**. Firstly, the higher soluble sugar concentration in grape than in tomato is a result of higher sugar importation, while metabolism and water dilution were the same in both fruit species. Secondly, the higher soluble sugar concentration in grape than in peach is a result of lower water dilution. The third mode of regulation is more complicated than the first two, with differences both in sugar importation and water dilution (grape vs. cherry tomato; cherry tomato vs. peach; peach vs. tomato). In this mode, a higher sugar importation was always followed with a higher water dilution (grape vs. cherry tomato; peach vs. tomato), and *vice versa* (cherry tomato vs. peach). Therefore, the relative extent of differences in water dilution and sugar importation led to a higher soluble sugar concentration. A fourth potential mode, namely a higher sugar importation with a lower water dilution, was not observed in the present dataset.

### PCA of Genotypes and Growing Conditions

**FIGURE 5:**
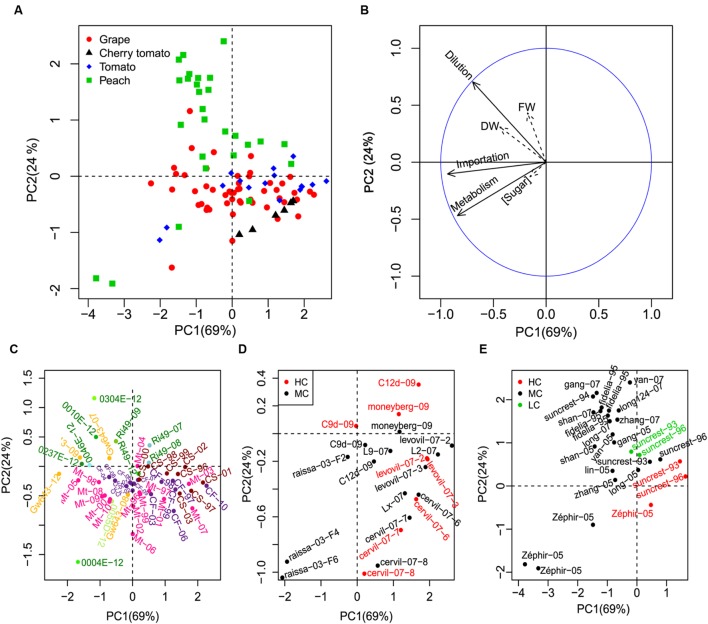
**Principal component analysis (PCA) analysis of genotypes and growing conditions of the three fruit species.** The three components (importation, metabolism, dilution) were used to make the PCA discriminate the three fruit species **(A)**. Soluble sugar concentration, FW, and DW were projected as non-active variables on the first two PCs **(B)**. To have a better view of the genotypes and growing conditions, a zooming of the general scatter plot **(A)** was conducted for each fruit (C for grape, D for cherry tomato and tomato, and E for peach). The genotype, year, and truss (for tomato) of the fruits were labeled as “genotype-year-truss.” Mt = Merlot, CS = Cabernet-Sauvignon, CF = Cabernet franc, GW643 = Gewurztraminer, Ri49 = Riesling in **(C)**. Green and yellow dots represent white grape genotypes and pink, violet and brown dots represent red grape genotypes **(C)**. HC, MC, and LC represent high, mean and low crop loads, respectively **(D,E)**.

In addition to the inter-species variability, genotypic and environmental variability was further analyzed by PCA. Mean cumulative values of sugar importation, metabolism and dilution were used to discriminate different genotypes and growing conditions (**Figure 5**). Results are plotted on the first two axes, which account for more than 90% of variability. The first axis mainly describes the effect of sugar importation and metabolism, whereas the second one deals with water dilution. Results confirm a reduction of import and, to a less extent, metabolism for cherry tomato, although there is a common tendency in large tomato too, as shown in **Figure 3**. For all species, a strong genotypic and environmental variability is present, especially along the first principal component (metabolism and sugar importation).

A closer look to individual genotypes and growing conditions, for each species, shows that PCA was able to discriminate a few phenotypic classes. White and red grapes were well-separated, with white grapes being characterized by an increased dilution term and a higher importation rate (**Figure 5C**), which is consistent with their larger fresh mass. Red grapes showed a high variability in the metabolic and import component (PC1), with the Cabernet-Sauvignon generally being the less sweet due to low import. Moreover, different genotypes showed varying environmental sensitivity, with Merlot being the most sensitive one to vintages in comparison with Cabernet-Sauvignon and Cabernet franc (**Figure 5C**).

Crop load exerted its effect on soluble sugar concentration using dilution and sugar importation as the main lever in peach (**Figure 5E**). A stable gradient of dilution was visible for Suncrest genotype conducted under three levels of load, in two different years. Interestingly, the dilution effect was stronger at low load but carbon content (and soluble sugar concentration) tended to be higher, meaning that carbon import increases faster than dilution. The same is true for the nectarine Zephir, although in this case the effect on dilution is accompanied by a strong change in sugar importation (**Figure 5E**).

In tomato, as in peach, crop load reduced fruit fresh mass but the mechanism may differ according to genotypes (**Figure 5D**). In cherry tomatoes, the impact of crop load was small and essentially acted by increasing slightly the metabolism. In large fruit genotypes, on the contrary, the impact of load appeared more important (except for Levovil), increasing dilution or decreasing slightly the metabolism.

## Discussion

Inter-species variability in soluble sugar concentration of grape, tomato, and peach was investigated by using a process-based framework, which decomposes soluble sugar concentration into three potential drivers (sugar importation, metabolism, and water dilution). Various datasets were collected and the developmental profiles of FW, DW, and soluble sugar concentration represented well the characteristics of each species described in the literature (Ho et al., 1987; Coombe and McCarthy, 2000; Génard et al., 2003), providing a solid base for our inter-species comparison.

The dynamics of the three processes (namely sugar importation, metabolism, and water dilution) grouped non-climacteric grape and climacteric tomato fruits together and discriminated them from the climacteric peach fruits (**Figure 3**). This suggests that the three processes related to soluble sugar concentration are not tightly affected by ethylene-associated events that characterize the two categories of fruits. In fact, Klie et al. (2014) compared the dynamics of metabolite concentration over development in non-climacteric strawberry and pepper fruits as well as climacteric peach and tomato fruits, and they also found that some cultivars of tomato were grouped with non-climacteric fruits and the others with climacteric peach fruit. Despite differences in respiration burst at the onset of ripening, cherry tomato and tomato are different from grape and peach by accumulating transiently starches during early development stages, which are then degraded to form soluble sugars around maturity (Schaffer and Petreikov, 1997; Luengwilai and Beckles, 2009; Petreikov et al., 2009). The transient accumulation of starches in cherry tomato and tomato is most likely reflected by the much higher levels of both sugar importation and metabolism during the early developmental stages (10% maturity to 40% maturity). On the other hand, positive values of metabolism were observed around maturity in cherry tomato and tomato (**Figures 3B,C** and **Supplementary Figures S1B,C**), and they may be a result of the starch degradation when approaching maturity. It is also worth noting that a positive value of “water dilution” indicates a positive effect on soluble sugar concentration due to fruit dehydration and this was evident in most of grape berries (**Figure 3A** and **Supplementary Figure S1A**). In fact, grape berries are known to be vulnerable to dehydration around maturity, which concentrates soluble sugar concentration without necessarily modifying total sugar quantity in the berry (Tilbrook and Tyerman, 2009). Based on the dynamic analysis, it is clear that the process-based decomposition can capture inter-species features related to soluble sugar concentration.

Our analysis shows the existence of different patterns for soluble sugar concentration control, either import-based, dilution-based, or import-dilution coupled (**Figure 4**). On the other hand, conserved metabolic rate was observed among the three fruit species for the consumption of imported carbon for synthesis of other compounds than sugars (e.g., starch, organic acids, structural carbohydrates, and proteins). Sugar importation is well-regulated by sugar transporters and the sugar gradient between phloem and fruits (Lecourieux et al., 2014; Osorio et al., 2014). Jensen et al. (2013) has analyzed the phloem sugar concentration of 41 species reported in more than 50 experiments and estimated that the optimal concentration for sugar transport in plants is 0.235 g/g. The phloem sugar concentration was estimated to be 0.21 g/g for grape (Dai et al., 2008), 0.11 g/g for tomato (Liu et al., 2007), and 0.38 g/g for peach (Jensen et al., 2013). The lower phloem sugar concentration in tomato might be one potential cause of the lower sugar importation observed for cherry tomato and tomato (**Figures 3E–H**). In addition, more efforts are needed to compare the activities of sugar transporters among the three fruits to identify the underlying reasons of differences in sugar importation (Lecourieux et al., 2014; Osorio et al., 2014). Another noticeable aspect is that the mean cumulative contribution of each process is also affected by the developmental stage considered. If the early developmental stages were considered, cherry tomato and tomato showed very high levels of sugar importation in concert with high metabolism (**Supplementary Figure S2**). It will be interesting to quantify the relative contributions of the three processes in grape and peach during the early developmental stages.

Fruit water balance, which affects dilution, is a function of water influxes from xylem and phloem and water effluxes via skin transpiration (Fishman and Génard, 1998; Guichard et al., 2001; Dai et al., 2010). Fruit transpiration is related to environmental conditions (temperature and relative humidity) and skin water permeability that quantifies the permeation coefficient of the fruit surface to water vapor (Fishman and Génard, 1998). Skin water permeability varies largely amongst fruit species (Nobel, 1975), ranging from 26 cm/h for tomato (recalculated from Leonardi et al., 2000), 50–100 cm/h for grapes (Dai et al., 2008; Zhang and Keller, 2015), and 200–800 for peaches (Lescourret et al., 2001). Lescourret et al. (2001) assessed the effect of skin water permeability on peach fruit growth and found that low skin water permeability confers high WC in peach, which results in a higher dilution effect on soluble sugar accumulation. Surprisingly, we found that peach had a higher dilution component than grape, cherry tomato, and tomato (**Figure 3**), which seems to be the reverse of what can be extrapolated from the analysis of Lescourret et al. (2001). We postulate that differences in dilution among the three fruit species should originate from the water influxes. Therefore, phloem and xylem water conductivities of fruit species seem to be pertinent candidates for further comparative analysis.

Environments, growing conditions, and management practices may influence fruit growth and soluble sugar concentration, with different responses depending on species and genotype (Coombe, 1976; Nookaraju et al., 2010; Beckles et al., 2012; Kromdijk et al., 2014; Kuhn et al., 2014; Soltis and Kliebenstein, 2015). This variability was clearly shown in the PCA analysis of mean cumulative values of sugar importation, metabolism and dilution (**Figure 5**), confirming the analyses conducted in previous publications (Quilot et al., 2004; Dai et al., 2009; Prudent et al., 2011) where the data were collected. Among the variation factors, such as year, crop load, water supply, and genotype, the same genotypes were often clustered together. This highlights the importance of genotype on determining soluble sugar accumulation in fleshy fruits. Within a given genotype, we compared the contributions of sugar importation, metabolism and dilution in response to crop load modifications between peach and tomato (**Figures 5D,E**). Crop load manipulation is an effective way to modify the carbon balance between sources and sinks (Kromdijk et al., 2014). Its effect on sugar importation is rather straightforward, as observed in peach. However, not only sugar importation is modified, the dilution component is also largely affected. Higher importation occurs in parallel with higher dilution in peach under low crop load. Crop load altering fruit water relationship has been reported (McFadyen et al., 1996). This suggests, on the one hand, a strong coordination between carbon and water influxes into fruits (Ho et al., 1987; Fishman and Génard, 1998; Guichard et al., 2001). On the other hand, it highlights the importance of growing conditions on the metabolite patterns in each fruit species (Kromdijk et al., 2014; Soltis and Kliebenstein, 2015) and pinpoints out the necessity of considering dilution effect in metabolic analysis (Génard et al., 2014).

In addition, the results obtained in this study could be useful in agricultural application. By representing biological processes and dissecting a complex trait into processes more physiological relevant and stable over changing environments (Bertin et al., 2010), the decomposition approach has also been applied to assist QTL identification in relation to sugar levels in tomato fruit (Prudent et al., 2011), evidencing its valuable role in marker-assisted breeding. The inter-species comparison conducted in this study highlighted different control modes of sugar concentration in each species, providing clues for breeding strategies to obtain fruits with targeted sugar levels (Tohge and Fernie, 2015). Moreover, the intra-species variabilities among different cultivars could also provide valuable agricultural implementations. For example, the different sensitivities of grape cultivars to dilution and importation may help to select suitable cultivars sensitive to agronomical factors such as irrigation or fruit load.

## Conclusion

Our analysis shows the existence of different patterns for soluble sugar concentration control, either import-based, dilution-based, or shared. On the other hand, conserved metabolic rate was observed among the three fruit species for the consumption of imported carbon for synthesis of other compounds than sugars (e.g., starch, organic acids, structural carbohydrates, and proteins). These distinct modes appear to be quite species-specific, with dilution being the main lever in peach, but a strong genotypic variability is present when considering the intensity of the effect. Growing seasons and management practices can further explain genotypic variability within a given species. These results provide novel insights into the drivers of differences in soluble sugar concentration among fleshy fruits and further emphasize the importance of dilution. In addition, the process-based decomposition framework proves to be a suitable tool for conducting inter-species comparison, because of its capability to decompose complex traits and extract stable and conserved information. It can be complementary to the metabolic multispecies comparison of Klie et al. (2014). It should be noted that the comparison presented here mainly focuses on the late developmental stages (40% maturity to 100% maturity), and it warrants more efforts to cover the whole fruit developmental stages for the inter-species comparison. Moreover, the underlying mechanisms of sugar importation and water influxes deserve further investigation for inter-species comparison, for example, by coupling the observed developmental profiles with the virtual fruit model that describes the process of phloem sugar importation and xylem water transport (Lescourret and Génard, 2005; Génard et al., 2007, 2010).

## Author Contributions

MG, VB, and ZD designed and oversaw the research; HW, MG, VB, and ZD performed the research and analyzed data. ZD, VB, and MG drafted the manuscript; CL, NB, HG, FL, BW, and ED contributed to data collection; CL, NB, HG, BW, ED, EG, FL, and SD critically revised the manuscript. All authors read and approved the final manuscript.

## Conflict of Interest Statement

The authors declare that the research was conducted in the absence of any commercial or financial relationships that could be construed as a potential conflict of interest.
