# Supplementary material for: Inter-Species Comparative Analysis of Components of Soluble Sugar Concentration in Fleshy Fruits
Source: Front Plant Sci. 2016 May 19;7:649. doi: 10.3389/fpls.2016.00649 (PMC4872523; doi:10.3389/fpls.2016.00649)
Supplement: Supplementary file 1 [file Data_Sheet_1.PDF]

Inter-species comparative analysis of components of soluble sugar concentration in fleshy fruits

Zhanwu Dai[1,*], Huan Wu[1,] , Valentina Baldazzi[2], Cornelis van Leeuwen[3], Nadia Bertin[2], Hélène Gautier[2], Benhong Wu[4], Eric Duchêne[5], Eric Gomès[6], Serge Delrot[6], Françoise Lescourret[2], Michel Génard[2]

[1]INRA, University of Bordeaux, ISVV, UMR1287 EGFV, 33882 Villenave d'Ornon, France

[2]INRA, UR1115, Plantes et Systèmes de Culture Horticoles, 84914 Avignon, France

[3]Bordeaux Sciences Agro, ISVV, UMR 1287 EGFV, 33882 Villenave d'Ornon, France

[4]Institute of Botany, Chinese Academy of Sciences, Beijing 100093, P. R. China

[5] INRA, UMR 1131 SVQV, 68021 Colmar, France

[6]University of Bordeaux, ISVV, INRA, UMR1287 EGFV, 33882 Villenave d'Ornon, France

*** Correspondance:** Zhanwu Dai, UMR1287 EGFV, INRA, University of Bordeaux, ISVV, 210, Chemin de Leysotte,CS50008, 33882 Villenave d'Ornon, France.

email: zhanwu.dai@bordeaux.inra.fr

Keywords (5-8): dilution, fruit metabolism, grape, peach, sugar importation, tomato

Supplementary materials

Supplementary table 1. Summary of the datasets used and their sources.

Fruit	type	Genotype	Species	Year	Treatment	Truss position	Sources
Grape	Red variety	Cabernet franc	*Vitis vinifera* L.	1996-2010	-	-	Present study
		Cabernet-Sauvignon	*Vitis vinifera* L.	1996-2003	-	-	Present study
		Merlot	*Vitis vinifera* L.	1996-2010	-	-	Present study
		Gewurztraminer	*Vitis vinifera* L.	2007-2009, 2012	-	-	Duchêne et al., 2012
	White variety	Riesling	*Vitis vinifera* L.	2007-2009, 2012	-	-	Duchêne et al., 2012
		0004E	*Vitis vinifera* L.	2012	-	-	Duchêne et al., 2012
		0304E	*Vitis vinifera* L.	2012	-	-	Duchêne et al., 2012
		0055D	*Vitis vinifera* L.	2012	-	-	Duchêne et al., 2012
		0010E	*Vitis vinifera* L.	2012	-	-	Duchêne et al., 2012
		0046E	*Vitis vinifera* L.	2012	-	-	Duchêne et al., 2012
		0237E	*Vitis vinifera* L.	2012	-	-	Duchêne et al., 2012
Tomato	cherry tomato	Cervil	*Solanum lycopersicum* var. *cerasiforme* (Dun.) Gray	2007	HC, LC	6, 7, 8	Bertin et al., 2009
	tomato	Levovil	*Solanum lycopersicum L.*	2007	HC, LC	2, 3	Bertin et al., 2009
		L2	*Solanum lycopersicum L.*	2007	-	-	Bertin et al., 2009
		L9	*Solanum lycopersicum L.*	2007	-	-	Bertin et al., 2009
		Lx	*Solanum lycopersicum L.*	2007	-	-	Bertin et al., 2009
		C9d	*S. lycopersicum* × *S. chmielewskii*	2009	HC, LC	-	Prudent et al., 2009
		C12d	*S. lycopersicum* × *S. chmielewskii*	2009	HC, LC	-	Prudent et al., 2009
		Moneyberg	*Solanum lycopersicum* L.	2009	HC, LC	-	Prudent et al., 2009
		Raissa	*Solanum lycopersicum L.*	2003	-	2, 4, 6	Present study
Peach	peach	Fidelia	*Prunus persica* (L.) Batsch	1995	-	-	Lobit et al., 2003
		Suncrest	*Prunus persica* (L.) Batsch	1993, 1994, 1996	HC, MC, LC		Génard et al., 2003

	Gang (Gangshanbai)	*Prunus persica* (L.) Batsch	2005, 2007	-	-	Wu et al., 2012
	Lin (Linbai 7)	*Prunus persica* (L.) Batsch	2005	-	-	Wu et al., 2012
	Long (Long 246)	*Prunus persica* (L.) Batsch	2005, 2007	-	-	Wu et al., 2012
	Long124	*Prunus persica* (L.) Batsch	2007	-	-	Wu et al., 2012
	Shan (Shanyibaitao)	*Prunus persica* (L.) Batsch	2005, 2007	-	-	Wu et al., 2012
	Yan (Yanhong)	*Prunus persica* (L.) Batsch	2,007	-	-	Wu et al., 2012
	Zhang (Zhanghuang 7)	*Prunus persica* (L.) Batsch	2005, 2007	-	-	Wu et al., 2012
nectarine	Zéphir	*Prunus persica* (L.) Batsch var. nucipersica (Suckow) C.K. Schneid.	2005	HC, LC, WS	-	Gibert et al., 2007

Note: treatments are mainly related to crop load, with HC for High crop load, MC for medium crop load, and LC for low crop load. WS indicates water stress treatment.

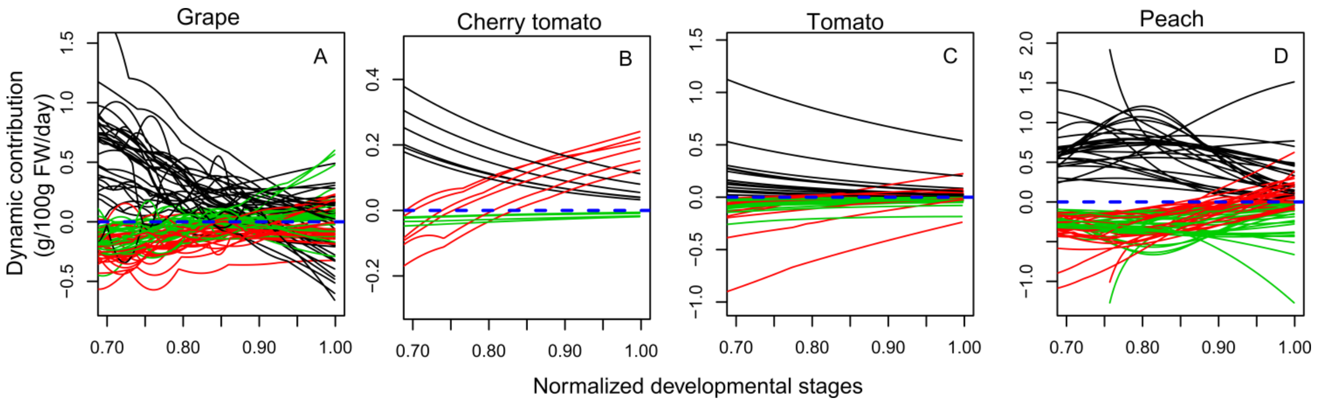

Supplementary Figure S1. The dynamic contributions of sugar importation (black), sugar metabolism (red), and water dilution (green) on sugar accumulation in grape, cherry tomato, tomato, and peach during the late fruit development stages (70% of maturity to 100% of maturity). The dashed blue lines indicate the zero at y axis.

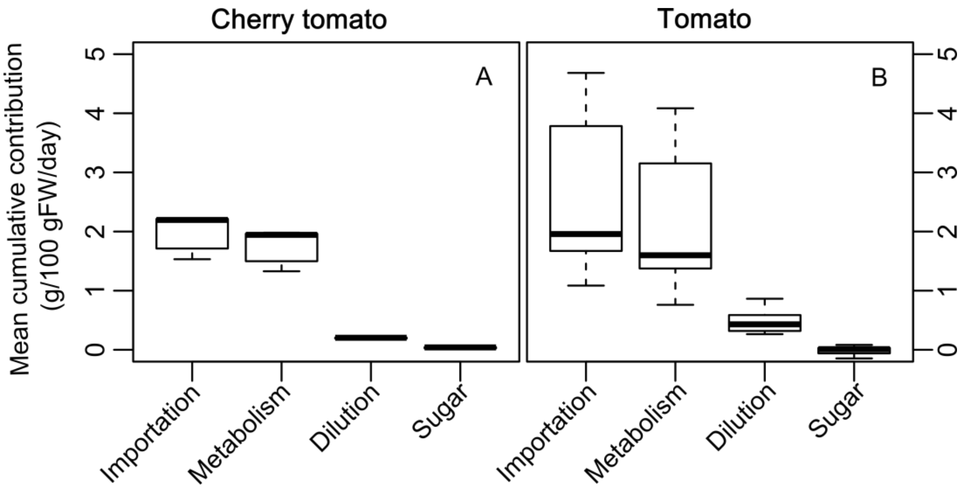

Supplementary Figure S2. The absolute value of mean cumulative contributions of sugar importation, sugar metabolism, and water dilution on sugar accumulation in cherry tomato and tomato during the early development stages.

To make the developmental profiles comparable among fruits, development stages were normalized with flowering to be 0 and maturity to be 1. Cumulative contribution was calculated over the period from flowering (10%) to 40% maturity, and divided by the duration (days) of the chosen period for each condition. Sugar represents the mean increment of sugar concentration during the targeted period.
